# New Perspectives to Improve Mesenchymal Stem Cell Therapies for Drug-Induced Liver Injury

**DOI:** 10.3390/ijms23052669

**Published:** 2022-02-28

**Authors:** Fernando Ezquer, Ya-Lin Huang, Marcelo Ezquer

**Affiliations:** Centro de Medicina Regenerativa, Facultad de Medicina, Clínica Alemana, Universidad del Desarrollo, Avenida la Plaza 680, Las Condes, Santiago 7610658, Chile; eezquer@udd.cl (F.E.); yhuang@udd.cl (Y.-L.H.)

**Keywords:** mesenchymal stem cells, drug-induced liver injury, secretome, exosomes, cell therapy

## Abstract

Drug-induced liver injury (DILI) is one of the leading causes of acute liver injury. Many factors may contribute to the susceptibility of patients to this condition, making DILI a global medical problem that has an impact on public health and the pharmaceutical industry. The use of mesenchymal stem cells (MSCs) has been at the forefront of regenerative medicine therapies for many years, including MSCs for the treatment of liver diseases. However, there is currently a huge gap between these experimental approaches and their application in clinical practice. In this concise review, we focus on the pathophysiology of DILI and highlight new experimental approaches conceived to improve cell-based therapy by the in vitro preconditioning of MSCs and/or the use of cell-free products as treatment for this liver condition. Finally, we discuss the advantages of new approaches, but also the current challenges that must be addressed in order to develop safer and more effective procedures that will allow cell-based therapies to reach clinical practice, enhancing the quality of life and prolonging the survival time of patients with DILI.

## 1. Drug-Induced Liver Injury, a Condition That Requires New Therapeutic Alternatives

Drug-induced liver injury (DILI) is defined as an injury to the liver caused by multiple drugs, herbs or other xenobiotics leading to abnormalities in liver tests or liver dysfunctions following the reasonable exclusion of other etiologies [1]. Despite the increasing awareness of hepatoxicity and the availability of less toxic drugs, the absolute frequency of hepatic drug reactions does not decrease, in keeping with the increasing number of medical prescriptions and the availability of a broad spectrum of pharmacological agents. Hepatic drug reactions are one of the leading causes of acute liver injury, accounting for 13% of cases of acute liver failure (ALF), and have become a global medical problem, particularly for public health systems and the pharmaceutical industry [2,3,4].

Many factors may contribute to the susceptibility of a patient to DILI, such as underlying chronic liver disease, viral infections and alcohol intake [2]. A number of studies have demonstrated that patients with obesity and/or nonalcoholic fatty liver disease (NAFLD) have impaired liver regeneration [5], which markedly increases their risk of developing DILI [6]. Therefore, obese patients are more prone to suffer DILI due to their overconsumption of several drugs and the intrinsic susceptibility of their diseased liver [7,8].

**Figure 1:**
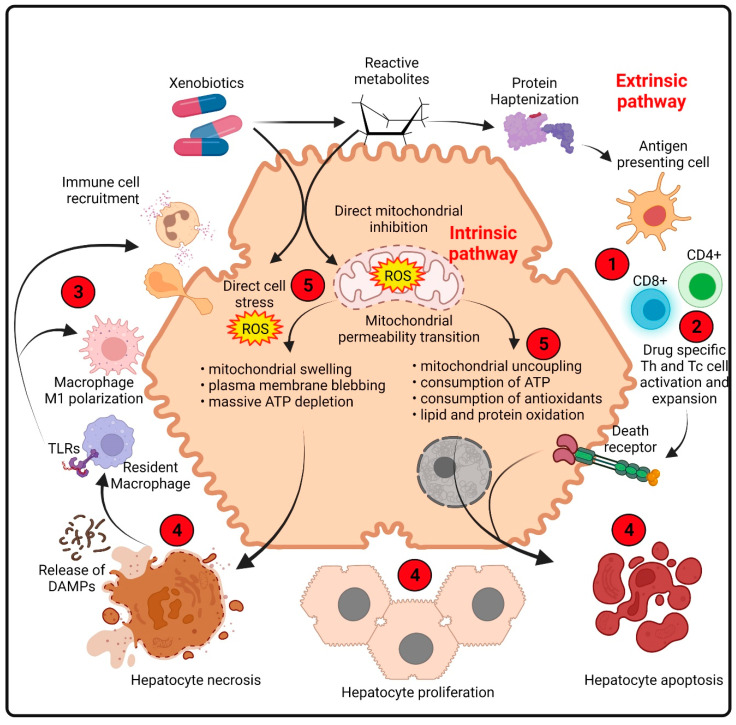
Pathogenesis of drug-induced liver injury. The normal hepatocyte shown in the center of the figure may be affected in multiple ways, including mitochondrial impairment [9], reactive metabolites that induce chemical and oxidative stress and protein modifications (both by intrinsic pathways) and innate and adaptive immune responses (extrinsic pathways) [18]. The impairment of the energetic and redox balance finally triggers apoptotic or necrotic processes according to poor or insufficient ATP levels [19]. Drugs are relatively small molecules and, therefore, are unlikely to evoke an immune response. However, they can act as haptens and bind covalently to proteins [20]. The resulting adducts are taken up by antigen-presenting cells (APCs) and processed into peptides, which are then presented to CD8+ and CD4+ cells in association with MHC class I or II molecules, respectively [21]. Apoptosis occurs in concert with immune-mediated injury, destroying hepatocytes by way of the activation of the tumor necrosis factor-alpha (TNF-α) receptor and Fas pathways, with cell shrinkage and fragmentation of nuclear chromatin [19]. The red circles indicate the DILI pathological events that may be moderated by the administration of MSCs (see Figure 2). DAMPs: damage-associated molecular patterns; ROS: reactive oxygen species; TLRs: toll-like receptors. Created with BioRender.com; accessed on 23 February 2022.

*Proposed pathogenesis:* Some key mechanisms have been associated with the onset of DILI and are summarized in Figure 1.

Certain xenobiotic molecules, either directly or after the metabolic release of intermediates, can cause mitochondrial alterations at different levels, affecting β-oxidation (resulting in decreased ATP production) and the respiratory chain enzymes. Free fatty acids cannot be metabolized, and the lack of aerobic respiration results in the accumulation of lactate and the generation of ROS within the organelle, which ultimately leads to the activation of the mitochondrial permeability transition and the release of mitochondrial proteins such as cytochrome C, and the activation of the caspase pathways [9].

In this context, nuclear factor erythroid 2-related factor 2 (Nrf-2), which regulates the synthesis of the antioxidant glutathione synthase (GSH), plays a key role in liver regeneration, as demonstrated in murine models of acetaminophen (APAP) intoxication in which the expression of the Nrf-2 gene has been reported to be reduced [10,11,12].

The evidence also supports a role for hepatic innate immune cells in tissue injury progression and severity in some cases of DILI, since a sterile inflammation can be initiated by the release of damage-associated molecular patterns (DAMPs) from necrotic cells and their recognition by toll-like receptors on macrophages [13].

These events trigger the release of cytokines and chemokines, activating and recruiting neutrophils and macrophages derived from monocytes into the necrotic areas [14].

In a complementary way, drug metabolism and the generation of reactive metabolites that bind to proteins, even in the absence of extensive cell death, can induce an adaptive immune response, which eventually also results in severe liver injury [15]. The additional tissue damage generates an abnormal overload of ROS, which results in cellular damage, triggering a vicious cycle. This drug-induced inflammation and ROS production are linked to organelle damage, which is another critical factor that leads to necrosis or apoptosis of hepatocytes in DILI [16].

*General treatment and specific therapies:* Once a liver injury occurs, the most important initial step in terms of disease management is to discontinue exposure to the noxious agent. Specific therapies can be applied to specific forms of DILI; for example, cholestyramine has been recommended to speed up drug clearance in leflunomide DILI induction, carnitine appears to be a specific antidote for valproate hepatotoxicity, and N-acetylcysteine is used in paracetamol intoxication [17]. However, if there is ALF, liver transplantation is the only successful treatment, whereas extracorporeal liver support may be an alternative treatment. However, these experimental approaches are only available to a limited number of patients [17].

## 2. Mesenchymal Stem Cells and Liver Regeneration

The lack of therapeutic alternatives for DILI requires the development of therapeutic agents capable of preventing further damage to the injured liver cells. Multipotent mesenchymal stromal cells also referred to as mesenchymal stem cells (MSCs), are a population of self-renewable and undifferentiated cells with great potential for the regeneration of damaged tissues, which are present in the bone marrow, adipose tissue, dental pulp and multiple other mesenchymal adult tissues [22].

According to the International Society for Cellular Therapy (ISCT), isolated and purified MSCs must meet three criteria: (i) adhere to plastic under standard culture conditions; (ii) present CD90, CD105 and CD73 markers, and lack hematopoietic and endothelial markers including CD11b, CD14, CD31, CD45, CD34; and (iii) differentiate into adipocytes, osteocytes and chondrocytes in vitro [23].

Systemically injected MSCs have been reported to travel through the bloodstream until they reach the damaged organs, establishing in the tissue parenchyma in a process known as “homing”. This process is mediated by the direct migration of MSCs upon activation of their CXCR4, CX3CR1 and CKR2 surface receptors by the SDF-1 and MCP-1 cytokines, whose genes are overexpressed in damaged tissues [24].

**Figure 2:**
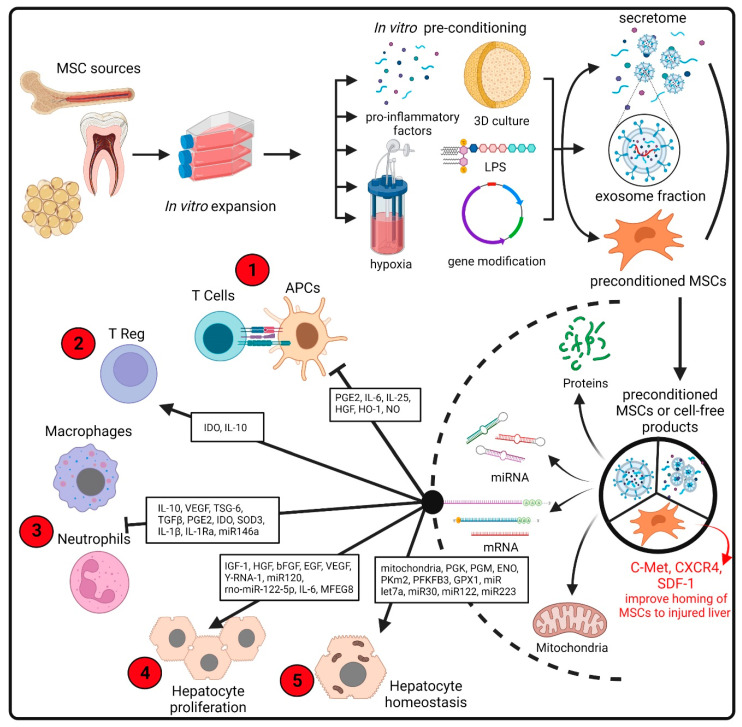
Comprehensive management of the production of improved MSCs for transplantation or cell-free products. Preconditioned MSCs or therapeutic products based on MCS secretome can be generated from stem cells isolated from common sources such as bone marrow, adipose tissue or dental pulps (among multiple sources). The cells are expanded in culture, followed by in vitro preconditioning (including treatment with cytokines, drugs, 3D cultures and hypoxic environment) to enhance the production of key molecules in the pathogenesis of liver disease. In addition to MSC pretreatment, gene modification is also used to improve the therapeutic effects of MSCs in liver diseases [63]. The secretome is subsequently collected and subjected to procedures intended to remove the cellular components. This secretome comprises soluble proteins and secreted extracellular vesicles. Both MSCs and cell-free products have a wide range of therapeutically beneficial effects mediated by the biological activity of a diverse range of proteins, lipids or RNA molecules [127]. The therapeutic properties include immunoregulatory activities on multiple innate and adaptive cells [28] and the secretion of a broad spectrum of angiogenic and mitogenic factors that stimulate hepatocyte proliferation [33]. Recently, the transfer of mitochondria from MSCs to damaged cells has been proposed [123]. The red circles indicate the DILI pathological events that may be moderated by MSC administration (see Figure 1). bFGF: basic fibroblast growth factor; CXCR4: C-X-C motif chemokine receptor 4; EGF: epidermal growth factor; ENO: enolase; GPX1: glutathione peroxidase 1; HGF: hepatocyte growth factor; HO-1: heme oxygenase 1; IDO: indoleamine 2,3-dioxygenase; IGF-1: insulin-like growth factor 1; IL1Ra: interleukin 1 receptor antagonist; LPS: lipopolysaccharide; MFG8: milk fat globule EGF factor 8 protein; NO: nitric oxide; PGE2: prostaglandin E2; PGK: phosphoglycerate kinase; PGM: phosphoglucomutase; PFKFB3: 6-phosphofructo-2-kinase/fructose-2,6-biphosphatase 3; PKm2: pyruvate kinase m2 isoform; SDF-1: stromal cell-derived factor-1; SOD3: superoxide dismutase 3; TGFb: transforming growth factor-beta; TSG-6: tumor necrosis factor (TNF)-stimulated gene 6; VEGF: vascular endothelial growth factor. Created with BioRender.com.

As summarized in Figure 2, MSCs may contribute to the regeneration of injured tissue through at least three mechanisms. (i) Differentiation into parenchymal cells: diverse studies have shown that MSCs derived from different sources can be induced to differentiate into a variety of cells, including hepatocyte-like cells [25]. (ii) Secretion of trophic factors that promote tissue regeneration: once in the damaged tissue, MSCs can sense the microenvironment and change their pattern of secreted biomolecules according to the special needs of the damaged tissue. These molecules include hepatocyte growth factor (HGF) and vascular endothelial growth factor (VEGF), which are crucial factors in the positive regulation of hepatocyte proliferation and liver regeneration [26]. In recent years, the microenvironment of injured tissue has been replicated in vitro and used to subject MSCs to a preconditioning stimulus to potentiate particular characteristics of these cells [27]. (iii) Modulation of the immune system. MSCs have been considered major actors in the regulation of both innate and adaptive immunity. Depending on the context, they can have an immunosuppressive or immune-stimulating capacity mediated by the secretion of soluble factors, like indoleamine 2,3-dioxygenase (IDO) and TNF-stimulated gene 6 protein (TSG-6), and by direct contact with immune cells [28].

MSCs can interfere with the maturation and antigen-presenting function of antigen-presenting cells (APCs), inhibiting T cell proliferation and inducing energy and decreasing CD8+ T cell cytotoxicity. Regulatory T cells (TReg) are necessary for the suppression of hepatocyte damage mediated by immune cells during ALF. MSCs significantly promote the proliferation of TRegs, thereby inhibiting immune cell activation [29]. Additionally, MSCs promote repolarization of macrophages from the M1 to the M2 phenotype characterized by high levels of IL-10 secretion, which can block the influx of neutrophils into the injured tissue and prevent further damage [30].

It is evident that, unlike conventional pharmacological treatments, MSCs act through diverse and complementary mechanisms. In this regard, multiple studies, including our own, have described the potential role of MSCs in promoting liver regeneration, even in special pathological conditions with impaired hepatic regeneration, like steatosis [31,32,33].

Promising early clinical proof-of-concept studies for the treatment of graft vs. host disease [34,35] and, more recently, for regenerative applications [36,37,38] have highlighted the safety of MSC-based therapies given the absence of serious adverse effects in treated patients. These results have supported the use of MSCs in the field of regenerative medicine and generated great expectations about the use of MSCs as a source of “multi-talented cells” for stem cell therapeutics, with broad clinical applicability. Indeed, the generation of a proregenerative microenvironment through the paracrine secretion of trophic factors is now the most accepted therapeutic mechanism associated with MSCs [39,40]. In fact, MSCs have been described as the “drug store” of the body [41].

However, the majority of the overwhelmingly positive results seen in preclinical studies have not translated into clinical efficacy [42,43]. The loss of a robust therapeutic response in inflammatory and degenerative diseases after the administration of MSCs has been largely attributable to the lack of cell engraftment or insufficient cell homing in the damaged tissue and/or to inadequate function in an inhospitable host environment [44].

Indeed, although the administration of MSCs effectively protects against liver injury in animal studies, it has not been as effective in well-designed clinical studies. Currently, there are 163 ongoing clinical trials using MSCs to treat different liver diseases (https://clinicaltrials.gov/; accessed on 13 December 2021). These studies focus mainly on two pathophysiological processes—liver cirrhosis and ALF. Little research has been conducted in other hepatic etiologies. There are no clinical trials centered on ALF derived from DILI. In the clinic, ALF progresses rapidly, having limited options of medical treatment, whereas liver cirrhosis is the end stage of progressive fibrosis, which lacks effective medical treatment. At present, liver transplantation is recognized as the most effective treatment for these conditions. However, the urgent clinical need is not met by the limited number of livers available for transplantation [45,46]. For these reasons, clinical trials have focused on these conditions and the priority of developing new therapeutic tools based on the use of MSCs.

The administration of MSCs showed only mild beneficial effects in decompensated cirrhosis, while in patients with ALF, MSC therapy was satisfactory in the short term [47,48,49].

It is important to note that the spectrum of etiologies related to these conditions is broad, including viral infections, autoimmune diseases, alcohol abuse and metabolic diseases. Likewise, marked heterogeneity is observed among studies regarding injection dose, cell source, delivery route and study design [50,51,52]. Therefore, the conjunction of multiple experimental protocols and the diversity in the etiology of liver diseases may lead to varying results in MSC therapy and derive in selection bias.

Since MSCs exhibit the strongest therapeutic effect during inflammation, they may be particularly effective in the context of acute, chronic liver failure (ACLF). The results of those studies show that MSC therapy is safe and that short-term efficacy is favorable, but survival was not markedly improved, generating controversy in terms of the benefits of MSC treatment [53,54,55,56,57].

In general, more attention should be given to large multicenter clinical trials, and well-designed randomized clinical trials are needed to verify the clinical effectiveness of long-term outcomes [57]. Additionally, more basic research is needed.

Recent studies have demonstrated that the regenerative ability of MSCs is not intrinsically determined and must be stimulated by signals from the local microenvironment [58,59]. The half-life of transplanted MSCs should also be considered inadequate for tissue regeneration, and in addition to having more effective cells, it is necessary to enhance post-transplant survival, one of the most important limiting factors of MSC-based therapies [60]. An early study showed that > 85% of the systemically injected MSCs are entrapped and lost in the lung precapillary vessels [61]. It has been postulated that MSCs exert tissue reparative and regenerative effects presumably through a brief “hit and run” mechanism, and thus long-term engraftment might not be a prerequisite to attain such effects [62]. Nevertheless, the initial survival of transplanted MSCs is a critical factor that commands the overall efficacy of MSC-based therapeutics.

Such evidence strongly indicates the need for further optimization of cell-based therapy approaches either by including specific cell pretreatment prior to transplantation, modifying the cell manufacturing process to obtain cell-free products or the combination of both.

## 3. Strategies to Improve the Regenerative Potential of MSCs

MSCs are characterized by their plasticity and can be greatly influenced by various extrinsic factors. Thus, in vitro preconditioning of MSCs is being explored in a variety of ways to enhance their therapeutic potential. Approaches to manipulate the MSC response include modulation of culture atmosphere, inflammatory cytokines, pharmacological agents, 3D culture, genetic modification and route of delivery [44,63] (Figure 2).

*Hypoxia*: In general, hypoxic preconditioning strengthens the regenerative and cytoprotective effects of MSCs by promoting the expression of genes encoding chemokine receptors or their ligands (CXCR4 and SDF-1) and subsequent in vivo engraftment [64,65].

According to Kojima et al., bone marrow MSCs cultured under hypoxic conditions showed greater therapeutic effects when transplanted in mice with liver cirrhosis. In this work, prostaglandin E synthase (PTGES) and miR120 were upregulated in hypoxic conditions. PTGES changed the macrophage phenotype to anti-inflammatory (M2), while miR120 reduced hepatocyte apoptosis [66]. Similarly, Yu et al. reported that MSCs grown under 1% oxygen highly expressed the VEGF gene and promoted liver regeneration in mice subjected to radical hepatectomy [67]. This functional enhancement of MSCs by hypoxia is not surprising, as their native environment in the bone marrow is hypoxic and plays an essential role in the regulation of their function [68].

*Inflammatory cytokines*: Given the natural interaction between the immune system and MSCs at the site of tissue injury/inflammation, culturing MSCs in a defined environment containing activating signals appears as a means to prime cells in order to increase their immunomodulatory activity [44,63]. In support of this approach, there are reports showing that only cytokine-activated MSCs, but not nonactivated MSCs, are effective in the reduction of an exacerbated inflammatory response [58]. Specifically, IL-6, PGE2, IDO and TSG-6 all seem to be major effector molecules in the immunomodulatory effects mediated by MSCs. The production of these potent immunomodulatory molecules is stimulated by proinflammatory cytokines such as IL-1β, TNF-α and INFγ [69]. The exposure of MSCs to INFγ stimulates IDO [70]; the pretreatment of MSCs with TNF-α increases migration, proliferation and angiogenic activity [71], whereas cytokine priming of MSCs with the combination of both TNF-α and INFγ can induce the secretion of cytokines such as IL-6, IL-5, IL-13, and increase the expression of the cyclooxygenase 2 (COX2) gene [72].

*Pharmacological agents*: alternative in vitro preconditioning of MSCs, includes the use of several pharmacological or chemical agents prior to their administration.

Given the fact that toll-like receptors (TLRs) found in MSCs recognize danger signals, agonists of these receptors have been evaluated to improve the regenerative function of MSCs [63]. Acceleration of the hepatic regenerative response and hepatocyte proliferation were enhanced by the use of secretomes obtained from MSCs stimulated in vitro with lipopolysaccharide (LPS) [73].

Deferoxamine (DFX) is an iron-chelating drug that stabilizes HIF-1α under normoxic conditions. Under this pharmacological pretreatment, MSCs increase the production of proangiogenic, proregenerative and anti-inflammatory factors [59]. DFX preconditioning also increases homing of MSCs by affecting chemokine receptors and metalloproteinases [74].

Some drugs that may serve as antioxidant agents can significantly improve the therapeutic effects of MSCs in eliminating liver fibrosis. Pretreatment with melatonin [75] or icariin [76] increase the homing ability and antioxidant activity of MSCs, improving hepatic fibrosis and accelerating the recovery of liver function.

Similarly, the combination of simvastatin and MSCs decreased hepatic collagen deposition and hydroxyproline content and impaired liver function by suppressing TGF-β/Smad signaling and production of α-smooth muscle actin in hepatic stellate cells (HSCs) [77].

*3D culture*: It is possible to consider three-dimensional cultures as an alternative for MSC preconditioning. MSCs growing in 3D spheroids are self-stimulated by autocrine IL-1β signaling, and this further enhances their anti-inflammatory effects [78]. Spheroid 3D cultures create a microenvironment where inner layers are exposed to lower levels of oxygen and nutrients, originating in a hypoxic environment. Compared to MSCs cultured in conventional 2D monolayers, MSCs growing in 3D spheroids up-regulate genes encoding factors associated with biological processes relevant to tissue repair, including angiogenesis [angiopoietin 2 (ANGPT2), basic fibroblast growth factor (bFGF) and VEGFa], antifibrotic effects [hepatocyte growth factor (HGF), transforming growth factor 3 (TGFβ3)] and anti-inflammatory effects [IDO, prostaglandin E2 (PEG2), TSG-6 and IL-10] [79]. Three-dimensional cultures of MSCs have also been shown to increase their antifibrotic potential since MSC spheroids were shown to decrease tissue fibrosis in a mouse model of hepatic cirrhosis [80].

Alternatively, the 3D culture induces a cytoskeletal reorganization that stimulates the expression of intracellular β-catenin and connexin 43, increasing the cell-cell interactions. Furthermore, integrin β1 is up-regulated in 3D cell structures, significantly increasing the cell-extracellular matrix adhesion relative to 2D monolayer cultures [81].

In addition, MSCs derived from spheroids are characterized by a significant reduction in cell size (to about 0.25–0.5 of the volume of an average cell from a monolayer culture), which could translate into fewer cells entrapped in the lungs after intravenous injection when compared to conventional MSCs from 2D cultures [82]. Moreover, MSC spheroids have been shown to have improved survival after administration compared to single-cell suspensions of MSCs [83].

The 3D culture could also influence the stemness of the MSCs. Thorp et al. reported that changes in the cytoskeletal structure stimulate mechanotransduction, mimicking early chondrogenic condensation by up-regulation of pro-chondrogenic signaling molecules as bone morphogenic protein 2 (BMP-2) and cartilage oligomeric matrix protein (COMP) [84].

*Genetic modifications*: Genetic modifications of MSCs can be employed to improve the therapeutic potency of MSCs independently of exogenous stimuli. Therefore, another approach to enhance the release of a specific regenerative factor is the overexpression of its gene in MSCs [85].

Overexpression of the gene encoding c-Met in MSCs derived from bone marrow improved their homing to the injured liver, thereby promoting the efficacy of MSC therapy for ALF [86]. Likewise, various studies have demonstrated that MSCs overexpressing the HGF gene present an increased ability to promote liver regeneration and suppress inflammatory responses [87,88].

MicroRNAs and other noncoding RNAs may reduce gene expression by targeting RNA for degradation and may determine the migration and therapeutic effects of MSCs. Overexpression of the gene for microRNA-122 does not alter the phenotype or differentiation potential of MSCs derived from adipose tissue in vitro, but effectively suppresses the activation of HSCs and eliminates collagen deposition in the liver, improving the therapeutic effects of MSCs [89].

Recently, advanced technology using CRISPR to achieve targeted genome editing facilitates detailed genetic editing at specific desired sites [90]. However, although stable and intensive potency can be guaranteed, genetic manipulation of MSCs is unfit to be applied to an actual application in the clinical field.

An important aspect that must be taken into account is the effect of in vitro preconditioning on the pluripotency of the cells. This point is directly related to the effectiveness of the therapy, but also bears relevance to its safety [91].

Hypoxic preconditioning has been shown to promote the multipotency of MSCs [92]. At the same time, it decreases the incidence of aneuploidy in MSCs compared to that in the normoxic condition, thus improving genetic stability and increasing the safety of MSC transplantation [93].

Two of the chemical agents mentioned previously, LPS and DFX can increase the survival and homing of transplanted MSCs. Preconditioning with LPS preserves the mitochondrial membrane potential and decreases the expression of the gene encoding connexin-43, thus stabilizing the cell membrane and preventing apoptosis of MSCs [94], while preconditioning with DFX increases the migration and homing of MSCs via increasing the production of HIF1-α, CXCR4 and metalloproteinases −2 and −9 [74].

In the same line, preconditioning MSCs with TNF-α enhances proliferation and osteogenic differentiation via the ERK1/2 and MAPK pathways [95] whereas the combination of TNF-α and IL-1β inhibits adipogenic and osteogenic differentiation, activating the canonical NF-κβ signaling pathway [96].

Obviously, these factors make the study of in vitro preconditioning more complex. However, they also represent an opportunity to design more powerful and safer therapies.

Although various MSC preconditioning strategies have been developed and have already been evaluated in multiple animal models, there are currently only three clinical trials registered (NCT01716481; NCT01849159; NCT02504437). All three use hypoxic preconditioning (with different protocols) to treat bone marrow MSCs in vitro prior to infusion, but none are being used for the treatment of liver diseases.

Another critical aspect in the design of MSC therapies is the delivery route. MSC transplantation can be applied via different routes, with varying efficiencies significantly improving the prognosis in experimental models of liver diseases.

*Routes of transplantation*: Intravascular MSC administration (via portal vein, hepatic artery or peripheral vein injection) is the most popular route for MSC administration in preclinical and clinical trials. Additionally, local administration routes such as intraperitoneal, intrasplenic and intrahepatic injection have been evaluated [27]. A current limitation for the design of clinical trials is the sparsity of studies that compare the therapeutic response under different methods of MSC transplantation.

Sun et al. showed that intravascular infusion was more effective than intraperitoneal injection in a model of ALF and that implantation in different blood vessels did not affect the transplantation outcome [97]. Comparing intravenous, intrahepatic and intraperitoneal injection routes, Zhao et al. demonstrated that intravenous injection was the most effective route to decrease the hepatic inflammatory cytokines (IL-1β, IL-6, TNF-α and TNF-β), reverse liver fibrosis and restore liver function [98].

In general, there is consensus that intravascular administration seems to be the best choice for MSC transplantation [24]. Moreover, in animal models of liver fibrosis, MSC administration in the tail vein had a therapeutic efficacy similar to that of intrahepatic injection [99]. However, the optimal route for MSC administration needs to be studied further to achieve the best effects in clinical trials. In addition to the transplantation route, the frequency of administration may be adapted to improve therapeutic efficiency. Miryounesi et al. showed that repeated infusions of MSCs (three times) significantly improved survival, liver fibrosis and necrosis when compared to infusion of MSCs in a single dose [100].

*Coadministration with scaffold materials*: Bioengineering incorporates scaffolding, an important contribution to the improvement of methods for MSC-based therapy. Bioactive reagents such as extracellular matrix and hydrogels are used to build 2D patches or organs using 3D printers. The biodegradable scaffolds can be loaded with cells and delivered to the target tissue, where the seeded cells can regenerate damaged tissue by cell migration and bioactive factor secretion [101]. In that sense, the use of scaffolds may increase the biophysical properties of MSCs, such as homing [102] and lineage determination [103].

Moreover, cell sheet engineering as a novel therapeutics in regenerative medicine is growing rapidly [101]. Many laboratories and biopharmaceutical companies have developed methods for the production of cell sheets based on new technologies, including temperature-responsive surfaces, photo-responsive materials, materials inducing ROS production, etc. [104]. In the same line, decellularized extracellular matrix (dECM) is considered an effective biomaterial in tissue engineering, providing a suitable physiological platform for engrafting and functionalizing the cells. Hepatic sheets for transplantation have been developed using hepatocyte-like cells derived from iPSc and MSCs incorporated in dECM scaffolds [105,106].

Preclinical studies using biocompatible materials have been actively conducted in recent years [107]. Studies have shown that the inclusion of MSCs in hybrid poly-(ethylene glycol)-alginate hydrogels protects them from entities such as circulating antibodies, as well as immune and inflammatory cells, without blocking nutrient and metabolite exchange, thus improving MSC efficacy in ALF and liver fibrosis models [108,109].

Recently, Ji et al. compared the cell proliferation and hepatic differentiation of MSCs in a biomatrix scaffold combined with growth factors from rat liver. In this experimental model, the scaffold stimulated the MSCs to express endodermal and hepatocyte-specific genes and to produce proteins associated with improved functions, and the cells exhibited the ultrastructural characteristics of mature hepatocytes, increasing the survival and liver function in CCl_4_-injured mice [110].

Alternatively, Kim et al. used hepatic progenitor cells obtained from patient biopsies as a new source for hepatic sheet transplantation. The functional analysis revealed the promotion of liver regeneration in a hepatic injury murine model by acquiring functional hepatocyte-like cells [111].

Several points need to be resolved: scaffold biomaterials could induce a prolonged host inflammatory response, reducing MSC survival and engraftment, and scaffolds physically isolate MSCs limiting endogenous autocrine and paracrine signaling and proper MSC phenotype. However, this is one of the most promising areas for the development of new therapeutic alternatives for liver diseases. In that sense, the potential benefits of engineered human cardiac muscle patch for the treatment of myocardial injury are readily observable in clinical studies [112,113].

## 4. Development of Cell-Free Therapies

The major constituents of the MSC secretome (also called conditioned medium) include a diverse range of soluble proteins and exosomes.

Exosomes are a family of nanoparticles with a diameter of 40–100 nm that are generated inside multivesicular endosomes or multivesicular bodies and secreted when these compartments fuse with the plasma membrane [114]. Exosomes are enriched in bioactive molecules such as proteins, lipids, mRNA and miRNAs [115] (more details on the molecular cargo and the extracellular signal transmission pathway of MSC exosomes are in the public domain at: www.exocarta.org; accessed on 13 December 2021; http://evepedia.info; accessed on 13 December 2021; and http://www.asemv.org; accessed on 13 December 2021).

The experimental effects observed with the use of the MSC secretome may result from either type of constituents—soluble molecules and exosomes—or both, and can be categorized under the following mechanisms: (i) antiapoptotic and/or antinecrotic effects and direct stimulation of cell proliferation and migration, (ii) immunomodulation and (iii) stabilization of the redox microenvironment [116].

The administration of the complete MSC secretome or only the exosome fraction, in in vitro and in vivo models of DILI, protects the hepatic cells from necrosis and/or apoptosis in the case of liver injury induced by APAP, H_2_O_2_ and CCl_4_ [117,118,119].

A single systemic administration of human MSC exosomes effectively reduced the levels of ROS and proinflammatory cytokines such as G-CSF, IL-1β, MCP-1, inhibiting oxidative stress and apoptosis in acute liver injury induced by CCl_4_ [120], and inhibited the apoptosis of hepatocytes induced by APAP and H_2_O_2_ through up-regulation of Bcl-Xl protein levels [119].

The increase in ROS induced by DILI impairs the synthesis of ATP and affects mitochondrial function and integrity. This condition is particularly evident when there is hepatic steatosis [6]. In this regard, Yang et al. showed that the systemic administration of MSC secretome reduced liver inflammation and enhanced its antioxidant capacity, increasing the expression of genes encoding sirtuin 1 (SIRT-1), peroxisome proliferator-activated receptor gamma coactivator alpha (PGC1a), Nrf-2 and mitochondrial transcription factor A (TFAM) maintaining mitochondrial function in an animal model of hepatic steatosis [121].

Intriguingly, all enzymes involved in the ATP synthesis coupled to glycolysis have been identified in MSC exosomes. Furthermore, oxidative stress was reduced via peroxiredoxins and glutathione S-transferases in MSC exosomes [122], which suggests that replenishing glycolytic enzymes through exosomal delivery to increase ATP production, as well as additional proteins to reduce oxidative stress, may help reduce cell death in the context of DILI physiopathology.

Actively transferring healthy mitochondria from MSCs can restore aerobic metabolism and protect cells from being eliminated [123]. Spees et al. showed in 2006 that MSCs could serve as mitochondrial donors in cell survival [124]. Mitochondrial DNA, mitochondrial fragments or entire mitochondria can be transported directly into the damaged site by MSC exosomes or macrovesicles [123,125]. This delivery can reduce apoptosis and increase cell viability by restoring the normal balance between aerobic respiration and glycolysis [125].

Compared to MSC transplantation, secretome therapy has several advantages: (i) using the secretome instead of the stem cells themselves decreases the risk of immune reactions and carcinogenesis; (ii) MSCs are efficient mass producers of secretome or exosomes, which can be manufactured in large scale, and stored to be used for liver regeneration at later time points; (iii) safety has been confirmed in vivo in multiple animal models and some clinical trials.

Given the diversity of proteins that are secreted from MSCs and the heterogeneity of vesicles that are released from them, it is unlikely that a unique factor having therapeutic value can be isolated. However, therapeutic products derived from the secretome would allow the enrichment of therapeutic factors and thereby maximize the therapeutic effect [116,126]. Therefore, the secretome, or the exosome subfraction, may represent ideal therapeutic tools for DILI in the near future.

Secretomes or exosomes were employed in four clinical trials currently underway (www.clinicaltrials.gov; accessed on 13 December 2021), although none of them are for the treatment of liver diseases: NCT04491240, NCT05019287, NCT05122234 for COVID-19 and NCT04134676 for chronic ulcer wounds. Scheduled to begin soon are eight new clinical trials: NCT04876326 for multiple system atrophy, NCT05008588 for subacute stroke infarct, NCT04326959 for keloid scars, NCT04314661 and NCT04223622 for osteoarthritis, NCT04889963 for ligament injury, and NCT046602442 and NCT04753476 for COVID-19, the latter with secretome from hypoxia preconditioned MSCs.

## 5. Limitations of Experimental Models of DILI and Strategies to Improve Them for Regenerative Medicine

The lack of precise experimental models that recapitulate the physiopathology of human DILI is one of the key points related to the lack of correlation between the preclinical data and the results observed in clinical trials and requires a separate section. To date, not a single model that accurately reproduces all aspects of human liver injury, including DILI, has been established [128,129].

The predominance of hepatocytes in the liver, in terms of abundance and functional contribution, has led to the use of primary human hepatocyte (PHH) cultures as the gold standard to test the therapeutic effects of MSCs in vitro. However, the use of these cells is greatly hindered because suitable human liver samples are scarce, show in vitro instability and high batch-to-batch functional variability related to huge variations in their content of cytochrome (CYP) P450 enzymes, which are responsible for their xenobiotic metabolic capacity and directly related to cytotoxicity. The mechanisms of cytotoxicity induced by APAP are very similar in PHH and livers of patients overdosed with APAP, which is not always the case for animal experimental models [130]. However, relatively high concentrations of APAP are needed to trigger toxicity in this in vitro setting compared to the concentrations found in the serum of patients with acute liver failure [131], limiting their use in routine testing [132].

Several hepatic cell models have been developed for drug metabolism and therapeutic screening to overcome these drawbacks. Hepatoma cell lines like HepG2, Fa2N4, Hep3B, Huh7 and HepaRG offer important advantages, such as their availability, unlimited life span and stable phenotype, which favor experimental robustness and reproducibility, and have been used to test MSC exosomes of in vitro DILI models [119]. In terms of detecting mitochondrial toxicity, some studies present HepG2 cells as more advantageous than PHH [133]. However, when compared to fresh human hepatocytes, HepG2 cells have low levels of CYP450 [134].

The HepaRG cell line is considered the closest surrogate of PHH for DILI applications and therapeutics testing since it reproduces hepatocyte-like characteristics, including more abundant CYP450 enzymes and higher content of bile acid transporters compared to HepG2cells [135]. However, their utility for toxicity screenings that involve large sets of compounds and their entire metabolism has not been sufficiently examined.

The in vitro approaches to study the therapeutic effects of MSCs on DILI can be controlled more closely than in vivo approaches. However, the selected conditions may not reflect those present in vivo well enough, like specific cell-to-cell interactions, the marked heterogeneity of enzyme activity of hepatocytes along the porto-central axis and the direct or indirect impact of drugs on other hepatic cells such as endothelial, Kupffer, stellate or biliary epithelial cells [128].

As mentioned for in vitro DILI models, drug metabolism, and especially metabolism mediated by CYP450, is a critical issue when working with any animal model of DILI. In that regard, although there are homologs of the CYP450 enzymes in all the species used as experimental animal models (mouse, rat, dog and monkey), not all homologs have the same substrate specificity [136].

Probably, one of the best models of DILI is the one resulting from an overdose of APAP in mice since the mechanisms of toxicity appear to be the same in mice and humans [137]. In contrast, rats are much less susceptible to APAP and develop only minor liver injury even at high doses [130]. These experimental data should be taken with caution since different mouse strains show variable sensitivity. Moreover, female mice are generally less susceptible to APAP than male mice [138], but there is no epidemiological evidence of lower susceptibility to APAP intoxication in female patients.

Significant progress has been made in the creation and use of humanized mice, although some methodological limitations still need to be resolved, including complications in the reproduction of standard chimeras, residual host immunoreactivity and a limited number of human cell types that can be used for xenotransplantation [128].

Complementary advances in in vitro 3D human cell culture systems have made it possible to study specific cellular functions in a liver-centered environment. Moreover, the field of stem cell research opened a lot of new roads with the isolation of iPSCs from humans, the combination with the new genome editing technologies, and the potential to identify the molecular and cellular bases of human diseases and therapies by “disease-in-a-dish” modeling [139]. Moreover, the decellularization and repopulation of the human liver represent the present and the future of hepatic tissue bioengineering, in which stem cell technology will have a critical role [140].

In this section, we focus on the need for experimental models of liver diseases and DILI, although the relevant issues are common to the rest of the diseases for which therapies based on the use of stem cells are sought.

Moreover, the emphasis should be placed on the development of assays that provide quantitative instead of simply qualitative results in order to define the classical parameters for any therapy, such as effective dose and therapeutic efficacy. While this is accomplished, the challenge is to collect relevant and sufficient information from as many models as are deemed necessary to make an informed decision regarding the potential benefits and risks to patients.

## 6. Challenges for Clinical Translation

Although the stem cell secretome (or exosome fraction) offers new promising perspectives for hepatic regenerative therapies, certain limitations persist that preclude the translation of these therapeutic approaches to the clinic for the treatment of DILI. The principal limitations are related to: *(i)* standardization of stem cell secretome composition; *(ii)* optimization of doses and routes of delivery; and *(iii)* unraveling the mechanisms of action of the stem cell secretome.

*I. Secretome composition*: the proteins or exosomes isolated from MSCs from different sources are highly heterogeneous, which may thus elicit different effects on their target cells. Moreover, the use of standardized and detailed protocols for growing and preconditioning cells will be necessary to ensure cell populations with uniform phenotypes and predictable behaviors. Indeed, when “the manufacturing process is the product”, as in the case of stem cell products, variability is the main enemy and must be reduced and controlled.

Cell culture variables include medium formulations (basal media and supplements), culture surface substrates, cell density and physicochemical environments, along with subculture protocols. It should also be considered that the isolation, large-scale production and characterization of exosomes have not been standardized [39,116,141,142].

Until a few years ago, the majority of studies incorporated fetal bovine serum (FBS) in basal media. However, concerns exist as FBS presents high variability in composition and may contain animal contaminants—which may pose risks for human recipients upon clinical translation.

Consequently, alternatives to FBS have been developed. These include humanized supplements like human serum or human platelet lysate (hPL). Human autologous serum has been reported to support human MSC expansion [143]. It would be problematic, however, to acquire enough serum to generate clinically relevant numbers of MSCs. Moreover, the use of autologous serum may not be applicable to elderly patients as its capacity to support cell growth may decrease with age [144].

hPLs have been reported to reduce immunological reactions and enhance stem cell culture expansion [145], and some data indicate that hPLs confer MSCs’ greater proliferative abilities than FBS, at least at early passage numbers [146].

Kim et al. compared the effect of hPL vs. FBS in MSC cultures [147]. They confirmed that MSC proliferation was enhanced when media were supplemented with hPL while the expression of genes encoding hepatoprotective factors like HGF and VEGF was similar in MSCs from cultures supplemented with either FBS or hPL. Interestingly, the cells cultured with hPL exhibited less attachment to the culture dish, probably related to the decreased expression of genes encoding actin, collagen I and laminin when compared to that of MSCs cultured with FBS.

It is important to note that hPLs are poorly defined and suffer from batch-to-batch variation, which can be a significant hindrance for the implementation of the production of MSCs at a clinical scale [148].

Serum-free media for MSC growth are a commercially available alternative. Nakashima et al. reported that the protein content of human adipose MSCs showed 98% similarity when cultured using FBS and a clinical-grade chemically defined medium [149]. However, most of these media have demonstrated only limited performance in supporting cell expansion for single passage cultures or at slow rates through multiple passages [148].

Once the appropriate cell source and culture protocol have been defined, one approach is to adopt the standard industry practice of establishing a master working cell bank; however, these are finite. An alternative approach could be the development of cell banks with immortalized MSCs or MSCs derived from iPSCs.

*II. Doses:* Unlike MSCs that are trapped mainly in the lung, exosomes derived from MSCs are predominantly found in the spleen and liver after systemic administration [150]. The difference between the in vivo fate of exosomes and that of living MSCs is related to their differences in biological characteristics, such as lipid composition and surface proteins.

The biodistribution and half-life analyses of exogenously administered exosomes are critical to determining the frequency and dose of injected exosomes. In the case of secretome administration, the risk of an immunogenic response may be increased when higher doses are used. Moreover, different studies have reported an optimal concentration of secretome to obtain therapeutic effects, while higher doses may have deleterious effects [151,152].

*III. Mechanisms of action:* The complete effects of the whole secretome of MSCs subjected to different preconditioning regimens have not yet been investigated in a comprehensive manner. As mentioned previously, the MSC secretome (or exosomes) contains hundreds of proteins and miRs, and many of the target genes could work independently, synergistically or even antagonistically.

Recent advances and cost reductions in high-throughput technologies, protein and RNA arrays and bioinformatics have already facilitated analysis of the secretome components and mechanisms of action and will continue to help in identifying the factors released by MSCs under different preconditioning regimens.

Another impediment associated with the translation of secretome and exosome preparations derived from MSCs into therapeutic products is the definition of potency metrics. As mentioned above, the problem arises from the complexity of the secretome (or exosome), which can potentially influence various biological processes.

In the present state of the art, there is a consensus in the scientific community that a full characterization of the mechanisms of action cannot be expected before the start of clinical evaluations and that an established potency standard may not be needed for clinical trials I/II [141].

Undoubtedly, one of the biggest challenges is the development and establishment of Good Manufacturing Practices along with standard protocols for MSC cell-free therapy. The ISCT Exosomes Scientific Committee has established the first guidelines to achieve this goal in the near future [153].

## 7. Conclusions and Perspectives

A large number of preclinical studies have demonstrated that MSCs exert therapeutic effects in liver diseases, including DILI, by promoting hepatocyte proliferation, regulating immunity and inhibiting fibrosis. However, in most cases, these therapies have not yet progressed beyond early phase clinical trials, and a huge gap between experimental approaches and their clinical applications is observed.

Currently, there are over 1000 registered clinical trials reporting the use of MSCs or their derivatives (secretome or microvesicles) (www.clinicaltrials.gov; accessed on 13 December 2021). Although more than twenty biotechnology companies are conducting phase II and phase III trials [154], none have met all the regulatory requirements to reach the clinic, even though 22 years have elapsed since the first patient received MSCs [155]. In fact, MSC therapy has only been approved for the treatment of acute GVHD in pediatric patients in Japan, Canada and New Zealand.

This can represent a discouraging scenario. Nevertheless, it may be very useful to make a comparison with the development of other therapies that represented breakthroughs in the medical field. Prockop et al. make a great and illustrating analogy with hematopoietic stem cell transplantation (HSCT), a procedure that is currently used in the treatment of more than 50,000 patients worldwide [156]. Success with this therapy is based on a series of discoveries that were made over more than six decades; clinical trials began in patients in the late 1950s, long before the basic understanding of the biology of hematopoietic stem cells was reached [157].

The early clinical trials with HSCT revealed problems that were not anticipated by the experiments in animals and re-steered the basic research. Moreover, this therapy has required continuous fine-tuning until the present [157].

The path to the clinic has been more complex for MSCs because the MSC therapy targets not just one tissue, such as bone marrow, but many organs and the specific diseases of these organs.

We believe that MSC therapy is in its initial steps in the sense that relevant data has been generated in animal models, but there is not a clear unifying hypothesis to explain the results, and there is no clear path to successful clinical trials. The way forward with MSCs requires overcoming some barriers.

One of the critical steps in developing a cellular therapy is to define as rigorously as possible the reagents and protocols that were used in the research. Since MSCs change dramatically in culture depending on the conditions of a large culture that include density, culture medium, exposure to oxygen, tissue origin, health and age of donor, an initial measure to address this problem is to persuade journals to require the complete data from the investigators.

MSCs represent a heterogeneous cellular population, defying a clear description. The criteria proposed originally by the ISCT [23] constitute a first step, but have well-known limitations since the epitopes used to define MSCs, although still among the best available, are not specific. Recently, additional nonclassical markers (i.e., CD200, CD273, CD274, CD36, CD248 and CD140b) that may potentially discriminate MSCs derived from adipose tissue from other cell types have been proposed to establish a novel criterion that may contribute to obtaining a more homogeneous production of clinical-grade MSCs [158].

Another critical point to resolve is the availability of MSCs to be used as a common reference between international laboratories [159]. This goal is not easy to achieve, given the heterogeneity of both the MSCs themselves and the culture protocols addressed previously. However, several possible sources of reference standards of MSCs have been proposed, including immortalized MSCs, extensively expanded MSCs and, more recently, MSCs derived from induced pluripotent cells [160].

Quantitative in vivo assays to test the efficacy of MSCs is one of the principal goals to achieve. However, the pleiotropic effects of MSCs on injured tissues, and the lack of animal models that recapitulate most of the pathological components of human diseases, make the development of suitable in vivo assays a major challenge. Another major step will be to provide biomarkers that can predict the in vivo efficacy of MSCs. Lee et al. proposed the expression of *TSG-6* and showed a strong correlation between the level of TSG-6 mRNA assayed in the cultured MSCs and their effectiveness in suppressing induced inflammation of the cornea [161]. In a similar way, an in vitro macrophage assay has been proposed to predict the in vivo anti-inflammatory potential of exosomes derived from MSCs [162].

A consensus in the shifting of MSCs’ therapeutic potential to their paracrine mechanism of action is being formed within the scientific community. Cell-free therapy and MSC preconditioning overcome some of the limitations associated with conventional MSC-based therapy and represent the next generation of therapeutic technology to be applied in DILI and other hepatic diseases. However, some concerns need to be addressed, including the standardization of protocols for MSC culture, in vitro preconditioning and MSC secretome production, since, as previously detailed, different signaling pathways are activated when different protocols are used. Understanding how each stimulus affects MSC behavior is crucial to validating both the safety and the disease-specific therapeutic potential.

Although a great deal of work is needed to comprehend the full range of mechanisms involved in MSC paracrine signaling upon preconditioning, some patterns are emerging. Preconditioning with hypoxia and 3D cultures seems to increase the production of cytoprotective, proregenerative and proangiogenic soluble factors, whereas priming of MSCs with inflammatory cytokines such as INFγ, IL-1β and TLR agonists mainly promotes secretion of chemokines [63,127].

Advances in high-throughput techniques and bioinformatics tools, in combination with extensive and public databases in the area, will aid in understanding the biological mechanisms behind in vitro preconditioning, which appears essential to carry out differential fine-tuning for each disease target.

Finally, clinical trials with MSCs are being conducted in many diseases for which it is difficult to use controls and in which the underlying etiologies are heterogeneous (including the broad spectrum of liver diseases). As mentioned above, great progress will be made in the field with the establishment of public websites that collect complete data on the transcriptomes, the manufacturing processes and other features of the MSCs that are administered to patients, as well as the results of clinical trials.

In conclusion, there are diverse ongoing studies that explore strategies to enhance MSC efficacy, including in vitro pretreatment and gene modification of MSCs and the extraction and application of the secretome (or exosomes) from MSCs.

To counteract the pathological events that occur in DILI (Figure 1) with the cellular and molecular mechanisms associated with the therapeutic effects of MSCs (Figure 2), the administration of preconditioned MSCs or MSC secretome should (i) reduce oxidative damage; (ii) increase hepatic regeneration; (iii) reduce the inflammatory response; and (iv) restore the energetic balance and ATP production in hepatocytes. The benefits are not yet assured, and the challenges that need to be resolved to develop curative therapies for DILI are diverse. Nevertheless, acquiring the vast amount of data regarding MSCs and the new experimental approaches that could be made accessible now may provide important tools for devising protocols that lead to the clinical scale production of MSCs, which should benefit a lot of patients.
